# Sensibility Comparison in Reduction Mammoplasties: Is Superomedial or Inferior Pedicle Better?

**DOI:** 10.7759/cureus.35248

**Published:** 2023-02-21

**Authors:** Mehmet E Yeğin, Ecem E Yeğin

**Affiliations:** 1 Plastic, Reconstructive and Aesthetic Surgery, Ege University Faculty of Medicine, Izmir, TUR; 2 Bioinformatics, Ege University Institute of Science, Izmir, TUR

**Keywords:** superomedial pedicle, inferior pedicle, symptom assessment, plastic surgery, mammaplasty, breast

## Abstract

Background

Breast reduction (BR) techniques have evolved tremendously since their introduction. However, a comparison of these techniques has yet to clarify the best choice or whether there is only one choice. This study aims to contribute to this research by retrospectively comparing the missing points of superomedial (SM) and inferior (INF) pedicle techniques.

Methodology

A total of 93 patients underwent surgery for gigantomastia with SM or INF. Demographic data, surgery details, and postoperative course data were obtained using the sixth-month two-point static discrimination test (TPD) of breasts. Patients were divided into SM and INF groups and statistically analyzed for differences in TPD and breast attributes, including suprasternal notch-to-nipple distance, ptosis grade, age, smoking history, parity, and body mass index.

Results

Intergroup analysis revealed significant differences between the SM and INF groups in TPD, with mean values of 21.03 ± 4.28 and 33.39 ± 6.91, respectively. The correlations between TPD results were tested, and only ptosis grades 2-4 and right suprasternal notch-nipple-areolar complex (NAC) distance were related.

Conclusions

The SM technique has better sensibility because the distance for transposition of the NAC to the newly designated position is shorter.

## Introduction

Breast reduction (BR) is one of the most common procedures performed in plastic surgery. Although the literature has standard endpoints of procedure mainlines, information about these methods is still evolving. One issue that remains a significant concern is the choice of the pedicle.

Inferior pedicle Wise-pattern BR (INF) is the most widely used method [1]. The primary concern with this technique is bottoming out of the inferior portion and lack of upper pole fullness in the long term. Lassus described the superomedial technique (SM), which resolved these concerns but had different problems [2]. With a similar rate of complications, SM was shown to be a better choice in patients who desire less reduction and have good skin and breast tissue density [3].

Another concern was the consideration of many patients, i.e., erogenous and touch sensitivity, essentially the nipple-areolar complex (NAC), after surgery. Sensory neurotization of the breast has been previously described [4]. The techniques suggested for measuring sensitivity varied among studies, including the Semmes-Weinstein monofilament test and two-point discrimination. The latter was advocated as a better and more objective method [5]. This study presents a similar comparison in a different population but with a more accurate method.

## Materials and methods

The local ethical committee approved this study (approval number: 21-1T/41; date: 15.01.2021). A total of 98 patient files operated on by a single surgeon between November 2017 and February 2020 were retrospectively examined. Patient records were scrutinized for a history of any previous operations in the thoracic area, age, obstetric history, smoking history, body mass index (BMI), bilateral suprasternal notch-nipple-areolar complex distance (R- and L-SSN-NAC), preoperative ptosis grade, operation reports, preoperative and postoperative photographs, complications, and results of the two-point static discrimination test (TPD) at the sixth month. Two patients were excluded due to previous operations in the thoracic area, and three were excluded due to insufficient documentation. According to the history and operation reports, the patients were divided into the SM and INF groups. The sixth postoperative month was considered the cutoff for neurotization of the operative area, and the TPD at the sixth-month visit was considered the main criterion for evaluating the outcomes.

SPSS version 25.0 (IBM Corp., Armonk, NY, USA) was used for statistical analysis. The Shapiro-Wilk test was used to determine the distributions. Parametric and non-parametric tests were used to calculate the mean and median values, respectively, to determine the relationship between the variables and TPD. Correlations were tested using Pearson’s test when a significant association was found between the groups. The statistical significance cutoff was set at p-values of 0.05.

## Results

In total, 67 patients underwent SM, and 26 underwent the INF pedicle technique. The mean ages were 33.49 ± 9.94 years and 41.85 ± 10.85 years in the SM and INF groups, respectively. A total of 51 patients had a history of smoking (54.8%), and 39 (41.9%) patients were nulliparous. The mean BMI values were 32.89 ± 2.25 kg/m^2^ and 35.22 ± 2.65 kg/m^2^ for the SM and INF groups, respectively. The mean SSN-NAC-R of breasts was 30.07 ± 2.38 cm in the SM and 31.61 ± 2.67 cm in the INF group. The mean SSN-NAC-L of breasts was 30.26 ± 2.68 cm in the SM and 31.40 ± 3.33 cm in the INF group. Ptosis grades were grade 2 in six and one patients, grade 3 in 48 and 11 patients, and Grade 4 in 13 and 14 in the SM and INF groups, respectively. TPD was 21.03 ± 4.28 mm and 33.39 ± 6.91 mm in SM and INF groups, respectively. Patient demographics are summarized in Table 1.

**Table 1:** Demographics of the patients. SM = superomedial pedicle, INF = inferior pedicle; BMI = body mass index; SSN-NAC (R) = suprasternal notch-nipple-areolar complex of the right breast; SSN-NAC (L) = suprasternal notch-nipple-areolar complex of the left breast

Group	Parameter	n	%	Parameter	n	%
SM (n = 67)	Age (years)			SSN-NAC (R) (cm)		
	19–29	27	40	≤30	38	57
	30–50	34	51	>30	29	43
	51–62	6	9	SSN-NAC (L) (cm)		
	Smoking history			≤30	37	55
	Yes	36	54	>30	30	45
	No	31	46	Ptosis		
	BMI (kg/m^2^)			2	6	9
	<35	52	78	3	48	72
	≥35	15	22	4	13	19
	Height (m)			Parity		
	<1.61	20	30	Nulliparous	33	49
	1.61–1.70	40	60	>1	34	51
	1.71–1.80	6	9			
	>1.80	1	1			
INF (n = 26)	Age (years)			SSN-NAC (R) (cm)		
	19–29	3	12	≤30	10	38
	30–50	17	64	>30	16	62
	51–62	6	24	SSN-NAC (L) (cm)		
	Smoking history			≤30	10	38
	Yes	15	58	>30	16	62
	No	11	42	Ptosis		
	BMI (kg/m^2^)			2	1	4
	<35	9	35	3	11	42
	≥35	17	65	4	14	54
	Height (m)			Parity		
	<1.60	6	23	Nulliparous	6	23
	1.60–1.70	18	69	>1	20	77
	1.70–1.80	2	8			
	>1.80	0	0			

Only two patients in the INF group had minor complications (wound dehiscence), and secondary intentional healing was sufficient. Eight patients in the SM group developed complications. One bilateral and one unilateral partial necrosis of the NAC was encountered, necessitating revision surgery. The remaining six patients healed with secondary intention without any further intervention.

**Figure 1:**
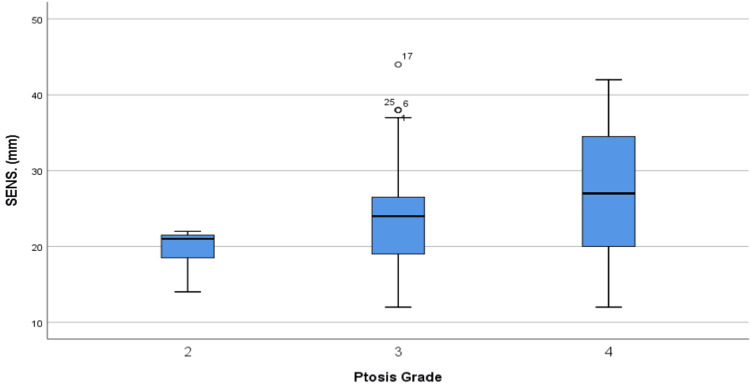
Boxplot of cases comparing patients’ sensitivity outcomes for different ptosis grades. The sensitivity outcomes of grade 2 and 4 patients differed significantly. SENS = sensitivity

TPD showed significant intergroup differences between the SM and INF groups with the independent-sample t-test (p < 0.01). Age and BMI showed weak correlations in the bivariate correlation test with TPD (p < 0.25). Among SSN-NAC values, only SSN-NAC-R in the INF group showed a moderate correlation with TPD (p = 0.31), and the others showed weak correlations (p < 0.25). Smoking showed statistically insignificant differences between the groups. A t-test was employed, in which smoking was found to affect TPD without significant differences (p > 0.05). Similarly, parity was found to be similar between the groups. A t-test revealed an insignificant difference in TPD between parous and non-parous patients (p > 0.05). Ptosis grades were non-normally distributed between the groups. The Kruskal-Wallis test was used to discover any significance, which resulted in a significant difference (p < 0.05). The post-hoc Bonferroni test was employed to reveal the ptosis grades that caused the difference, and intergroup differences between grades 2 and 4 showed significant differences in all patients (p < 0.05), respectively (Figure 1).

Complications had a non-normal distribution. The Kruskal-Wallis test revealed insignificant differences between the groups (p > 0.05).

## Discussion

This study focused on sensitive outcomes after BR using SM and INF techniques and revealed that SM is superior in sensibility. In addition, SSN-NAC-R and ptosis grades were other factors related to sensitivity return after the operation.

Some studies have tested demographic and physical features and complication profiles using these techniques. For example, Ogunleye et al. demonstrated that physical attributes or smoking do not change complication profiles, but age has a positive relationship [2]. Greuse et al. reported similar findings and interpreted their results as showing insignificant differences [5]. However, in this study, none of the parameters were strongly correlated with the complication rates.

Sensibility return (SR) is a common focus of comparative studies, in which the monofilament test is commonly used in BR techniques. Many neurological tests have been employed for this purpose, mainly the Semmes-Weinstein monofilament. However, it is a poor study technique with unpredictable outcomes [5,6]. Therefore, the TPD was determined to be a more objective parameter.

Many studies have discussed the role of preoperative breast size and the amount excised [2,6]. This study established a relationship between TPD, SSN-NAC distances, and ptosis grades. Furthermore, the SSN-NAC and ptosis grades estimate the tissue to be excised [4]. Therefore, the results of this study can be interpreted as the reduction would change the SR outcomes in favor of SM. However, selection bias in pedicle choice may have affected our outcomes. Therefore, it may be a wiser explanation to relate the distance for transposition of NAC with lower SR outcomes, in contrast to the study by Santanelli et al., which found that TPD was unrelated to distance [7].

All pedicle types under scrutiny for SR in BR were classified according to the nerves preserved during resection. However, the methodology of comparative studies to understand the relationship between pedicles and SR is misbuilt. Similar to our study, only the links between pedicle types and amounts of excised breast tissue or prior breast sizes have been focused on [1,2,8,9]. However, the excised skin, which bears nerve endings, has been overlooked. The excision amount of the skin may predominantly affect the receptor density and mislead observers, making the results vastly different among studies that research the same pedicle type. A surface analysis technique can be used in future studies for a more objective comparison.

Described pedicles for BR have clashed numerous times in the literature. Most recent studies suggest that SM is not superior to INF in terms of complications and SR. In this study, the comparison of SM and INF similarly resulted in a statistically significant difference for SR, while the complication comparison resulted in an insignificant difference. Interpretation of this analysis leads to the idea that SM is superior to INF in terms of SR, but patient characteristics remain controversial. The small study group may impair the precision of the study, which is a limitation of this study. In future studies, a large homogenous patient population, without less selection bias, can be discussed. Furthermore, previous skin characteristics and the amount of skin to be excised may add value to a future study.

## Conclusions

The pedicles used in BR have been compared in several studies. Most studies have suggested that superior-based pedicles are more physiological or have robust blood support. This study found that SM was superior to INF when SR was considered. However, our results suggest that there may be a bias between the pedicle choice and breast size. Therefore, studies that include the amount of excised breast and skin or compare similar anthropometric properties will help make a more sophisticated comparison in the future.
